# Analysis of Differentially Expressed Proteins Involved in Autoimmune Cirrhosis and Normal Serum by iTRAQ Proteomics

**DOI:** 10.1002/prca.201700153

**Published:** 2018-08-09

**Authors:** Zheng Minghui, Hu Kunhua, Bao Yunwen, Lu Hongmei, Li Jing, Wu Shaowen, Sun Longqiaozi, Duan Chaohui

**Affiliations:** ^1^ Department of Clinical Laboratory Sun Yat‐sen Memorial Hospital of Sun Yat‐sen University Guangzhou 510120 China; ^2^ Proteomics Center Zhongshan School of Medicine Sun Yat‐sen University Guangzhou 510080 China; ^3^ The Second Clinical Medical College Guangdong Medical University Dongguan 523808 China

**Keywords:** autoimmune cirrhosis, autoimmune hepatitis, biomarker, iTRAQ, LC‐ESI MS/MS

## Abstract

**Purpose:**

In order to study the candidate biomarkers in autoimmune cirrhosis (AIC).

**Experimental design:**

Isobaric tags are first implemented for relative and absolute quantitation technology on proteins prepared from serum obtained from AIC and normal controls. Proteins found to be differentially expressed are identified with liquid chromatography electrospray ionization tandem mass spectrometry by using a Q Exactive classic ion trap mass spectrometer.

**Results:**

108 proteins (32 upregulated and 76 downregulated proteins) are identified from AIC samples, compared with the normal controls. Gene Ontology enrichment analysis, KEGG pathway analysis, and protein–protein interaction map by STRING show that they associate with multiple functional groups, including ion binding activity, peptidase activity, and enzyme regulator activity. Finally, the von Willebrand factor, insulin‐like growth factor‐binding protein complex acid labile subunit, transthyretin, adiponectin proteins are identified with western blot as candidate biomarkers for AIC.

**Conclusions and clinical relevance:**

These findings offer a comprehensive profile of the AIC proteome about candidate biomarkers and provide a useful basis for further analysis of the pathogenic mechanism of AIC.

## 1 Introduction

Autoimmune hepatitis (AIH) is a global disease, which has a prevalence of 10–20 per 100 000 individuals and is seen more frequently among women (male to female ratio of 1:4). The peak incidence of disease occurs in the fourth or fifth decade of life.^1^ The etiology of AIH is not clear and is generally associated with heredity and environment.^2^ Two thirds of autoimmune cirrhosis (AIC) is mainly caused by chronic process of AIH, in which approximately one third of the adult patients and one half of the pediatric patients present with cirrhosis at diagnosis, and untreated patients generally advance to cirrhosis and liver failure.^3^, ^4^ Approximately one third of the patients have no clear history of hepatitis and are directly detected as AIC. Cirrhosis in AIH at any time during the course of disease is associated with a poor prognosis.^4^, ^5^ AIC can be developed into hepatocellular carcinoma or serious complications such as esophageal variceal bleeding, hepatic encephalopathy, which are harmful to people's life, bringing huge waste of medical resources and economic losses.^6^

The diagnosis of AIC disease, remains challenging,^7^ which mainly depends on medical history, clinical symptoms, and radiological examinations, but once AIC can be confirmed, AIC has reached the middle or late stages, leading to irreversible liver damage, missing the best time for therapy, forcing the patients for transplantation. However, the early damage of cirrhosis is reversible and the treatment effect is good which can effectively prevent the deterioration of the disease process.^8^ Therefore, AIC focuses on early diagnosis and early treatment, but the biochemical indicators were not specific, which are easily confused with AIH and is difficult to distinguish with other diseases, such as serum aminotransferase, high gamma globulin, autoantibody IgG positive, which can also be found in AIH, viral hepatitis, drug‐induced hepatitis, or other immune diseases.^9^ There is no specific diagnostic markers for AIC.

**Clinical Relevance**

Currently, the diagnosis of autoimmune cirrhosis (AIC) disease remains challenging, there are no specific diagnostic markers for AIC. In the present study, we first implemented isobaric tags for relative and absolute quantitation technique to identify the differentially expressed proteins in the serum of AIC compared to the normal controls. One hundred and eight differentially expressed proteins were identified and most of them were found to be involved in multiple functional groups, including ion binding activity, peptidase activity, and enzyme regulator activity. Moreover, we identified von Willebrand factor, insulin‐like growth factor‐binding protein complex acid labile subunit, transthyretin, adiponectin proteins as candidate biomarkers for AIC. These results are very helpful to elucidate the pathogenic mechanism of AIC.

Liver biopsy is still the most accurate and widely used method, by which cirrhosis can be diagnosed and staged. There are, however, notable disadvantages to this method of examination, including cost, risk of bleeding, and sampling error.^10^ So it is necessary to find specific biomarkers of AIC for early prevention, early diagnosis, and early treatment.

**Figure 1:**
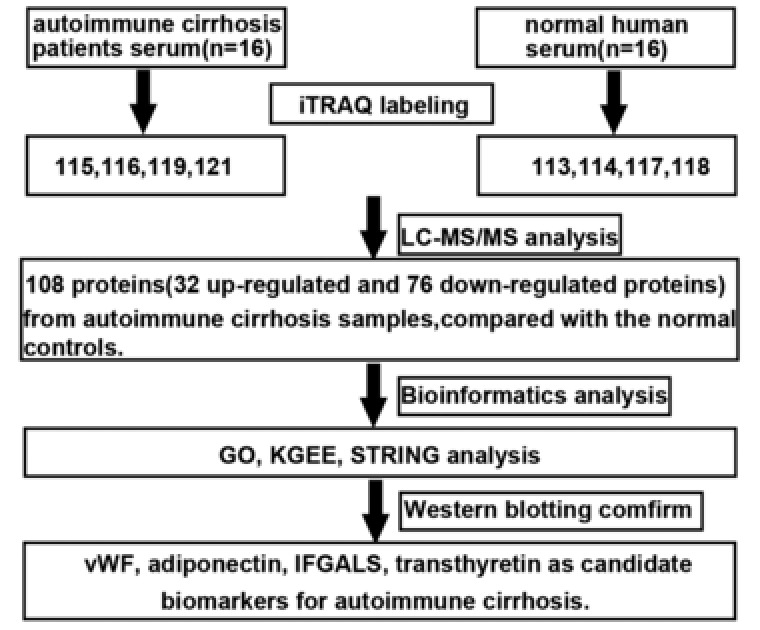
General work flow and summary of the present study. iTRAQ technology was applied to identify the differentially expressed proteins in autoimmune cirrhosis and normal controls. At the end of the study, we combined the results of bioinformatics analysis to identify vWF, adiponectin, IFGALS, and transthyretin proteins as candidate biomarkers for autoimmune cirrhosis.

Isobaric tags for relative and absolute quantitation (iTRAQ) technology has been currently used as a powerful tool for global evaluation of protein expression about specific diseases or disease stages, which shows significantly improved sensitivity and repeatability when compared with the traditional 2D electrophoresis , and has been widely applied in studying biomarkers for various diseases.^11^ In this study, the general workflow is shown in Figure 1, we aim to first identify potential serum proteins as biomarkers in AIC because of the convenience of serum samples. Bioinformatics analysis of differentially expressed proteins indicated that these proteins might have important roles in a variety of cellular processes and structures, including stress, immune system process, vesicle‐mediated transport, and signal transduction.

## 2 Experimental Section

*Preparation of Serum Samples*: Blood samples were collected from 16 patients (12 women and 4 men) with autoimmune cirrhosis diagnosed according to the following criteria^12^:1)excluding cirrhosis due to other causes; 2) AIH for at least half a year, or the clinical score reaching more than 15 points without AIH history; 3) B ultrasound indicated that the surface of liver was enhanced and thickened, not smooth or nodular, and the spleen enlarged or the vein widened; 4) hepatic hypofunction and 5) not treated with medication. The diagnosis of each enrolled patients was confirmed by liver biopsy. At the same time, patients were excluded if they had other diseases: 1) other cancers; 2) autoimmune diseases, such as rheumatoid arthritis, systemic lupus erythematosus; 3) severe infections; 4) basic diseases, such as diabetes, hypertension, coronary heart disease; 5) diseases of hematopoietic system, such as aplastic anemia, leukemi and 6) pregnancy. Another 16 matched (12 women and 4 men) blood samples from normal people were collected as controls. All the samples were provided by the Department of Infectious Diseases, Third Affiliated Hospital in Sun Yat‐sen University and separated by centrifugation at 800 × *g* for 30 min. Aliquots of serum were collected and stored at −80 °C. To reduce the individual differences, aliquots of serum samples from four randomly selected individuals in each group (AIC and healthy controls)were mixed to create eight pools. Groups n1, n2, n3, n4 (n = normal), p1, p2, p3, and p4 (p = patient) were formed.

Serum samples were processed using the ProteoPrep Blue Albumin Depletion Kit (Sigma, St. Louis, MO, USA), which selectively removes albumin and IgG from the serum sample according to the manufacturer's instructions. In order to depurate the protein extraction and determine the final protein concentration, the 2D Clean‐up Kit (GE Healthcare, UK) and 2D Quant Kit (GE Healthcare, London, UK) were used sequentially, following the manufacturer's instructions. Proteins were digested according to the FASP method.^13^ Protein samples (100 μg) were reduced using 2 μL of reducing agent and incubated at 60 °C for 1 h. Next, added 1 μL of cysteine‐blocking reagent at room temperature for 10 min using iTRAQ Reagent Multiplex Buffer Kit (AB Sciex, USA), and then transferred for ultrafiltration. Finally, 100 μL 1 m TEAB buffer was added and centrifuged at 12 000 × *g* for 20 min for three times. Trypsin (1 μg μL^−1^, 1:50, Progema, USA) was added to the samples for incubating at 37 °C for 16 h.

*Protein Labeling with iTRAQ*: iTRAQ labeling was performed according to the manufacturer's instructions (Applied Biosystems Sciex, #4381664). Groups n1, n2, n3, n4, p1, p2, p3, and p4 were individually labeled with iTRAQ reagent (including n1‐iTRAQ 113 reagent, n2‐iTRAQ 114 reagent, n3‐iTRAQ 117 reagent, n3‐iTRAQ 118 reagent, p1‐iTRAQ 115 reagent, p2‐iTRAQ 116 reagent, p3‐iTRAQ 119 reagent, and p4‐iTRAQ 121 reagent).The labeled samples were allowed to incubate at room temperature for 2 h, then all the serum samples were mixed together and vacuum dried.

High pH Reverse Phase Fractionation: High pH reverse phase fractionation chromatography was carried out using a Dionex UltiMate 3000 HPLC system. The iTRAQ‐tagged peptides were reconstituted and loaded onto Gemini‐NX C18 columns (3 μm, 2 × 150 mm, 110 A, Phenomenex). The peptides were eluted with a linear gradient formed by buffer A (20 mm NH4HCO2, pH = 10) and buffer B (20 mm HCOONH4, 80% acetonitrile, pH = 10) for 100 min at a flow rate of 200 μL min−1. Twenty‐four fractions were collected at 1 min intervals, based on UV detection wavelength at 214/280 nm. Then all the fractions were dried individually and dissolved in the sample solution (0.1% formic acid and 2% acetonitrile) for nano LC–MS /MS analysis.

*LC–MS/MS Analysis by Q Exactive*: The nano LC–MS/MS was carried out using Q Exactive (Thermo Scientific) with the peptide recognition mode enabled. The peptide mixture was separated on the chromatographic column (75 um × 150 mm, packed with Acclaim PepMap RSLC C18, 2 μm, 100A) at a flow rate of 300 nL min−1. And then peptides were eluted from the HPLC column through the application of a linear gradient from 4% to 50 % solution (0.1% FA, 80% ACN) for 40 min. Finally, the eluted peptides were detected by Q Exactive and MS data were acquired using a data‐dependent top 20 method, dynamically choosing the most abundant precursor ions from the survey scan (350–1800 m/z, resolution of 70 000 at m/z 200) for high‐energy collisional dissociation (HCD; resolution of 17 500 at m/z 200, collision energy 30 eV) fragmentation with each component analysis for 60 min.

*Protein Identification and Data Analysis*: First, the raw files were converted to .mgf files by Proteome Discoverer 1.4 (Thermo Fisher Scientific). Then, Protein Pilot 5.0 (AB Sciex, USA) was used for protein identification and quantification analysis. Database searching parameters were as follows. Sample type: iTRAQ 8plex (Peptide Labeled), Cys alkylation: MMTS, ID focus: biological modification, digestion: trypsin, Search effort: thorough ID, FDR <1%, T‐test was used to identify significant differences (p < 0.05) in means between AIC and control with an average ratio‐fold change ≥1.5 or ≤0.66, a minimum of two peptide matches in common was confidently considered as differential expression of proteins. The coefficient of variation was used to evaluate the dispersion of the replicates within groups, and detected proteins with coefficient of variation ≤ 0.5 were considered reliable.

Bioinformatics Analysis: Bioinformatics analysis was carried out in order to better study the biological function of significantly altered proteins. GO annotation and enrichment analysis (http://www. geneontology.org) which includes three main modules—biological process, cellular component, and molecular function—was employed to categorize proteins into families and subfamilies with shared functions. Pathway analysis using KGEE (http://www.genome.jp/kegg/pathway.html) was used to take advantage of the current knowledge of biochemical pathways and protein–protein interaction networks using STRING database (http:// www.string-db.org) which is a database of known and predicted protein interactions, including direct (physical) and indirect (functional) associations.

Western Blot Analysis: Serum samples of the same amount of protein were separated by SDS‐PAGE and analyzed by western blot. In brief, proteins (60 ug) in the gels were transferred onto PVDF membranes (Roche). After blocking for nonspecific binding with 5% non‐fat milk, the membranes were incubated with primary antibodies against apoE (abcam, #7613), apoA1 (abcam, #7620), von Willebrand factor (vWF; CST, #65707), insulin‐like growth factor‐binding protein complex acid labile subunit (IFGALS; abcam, #85222), gelsolin (abcam, #109014), adiponectin (CST, #2789), transthyretin (TTR; abcam, #92469), pIgR (abcam, #96196) at 4 °C overnight, followed by the appropriate horse‐radish peroxidase‐conjugated secondary antibodies. All blots were visualized using ECL (Amersham Biosciences).Quantification was performed using ImageJ software.

*Statistical Data and Graphics*: The statistical analyses were performed using GraphPad Prism version 6.01 for Windows (GraphPad Software, La Jolla, California, USA; www.graphpad.com). Student's t‐test was applied for comparisons of quantitative data. The receiver operating characteristic (ROC) analysis with SPSS Statistics 20.0 was performed to evaluate sensitivity and specificity of each marker and their combination in AIC and AIH. For all analysis, p value < 0.05 was considered to indicate statistical significance.

## 3 Results

### 3.1 Primary Data Analysis and Protein Identification

**Figure 2:**
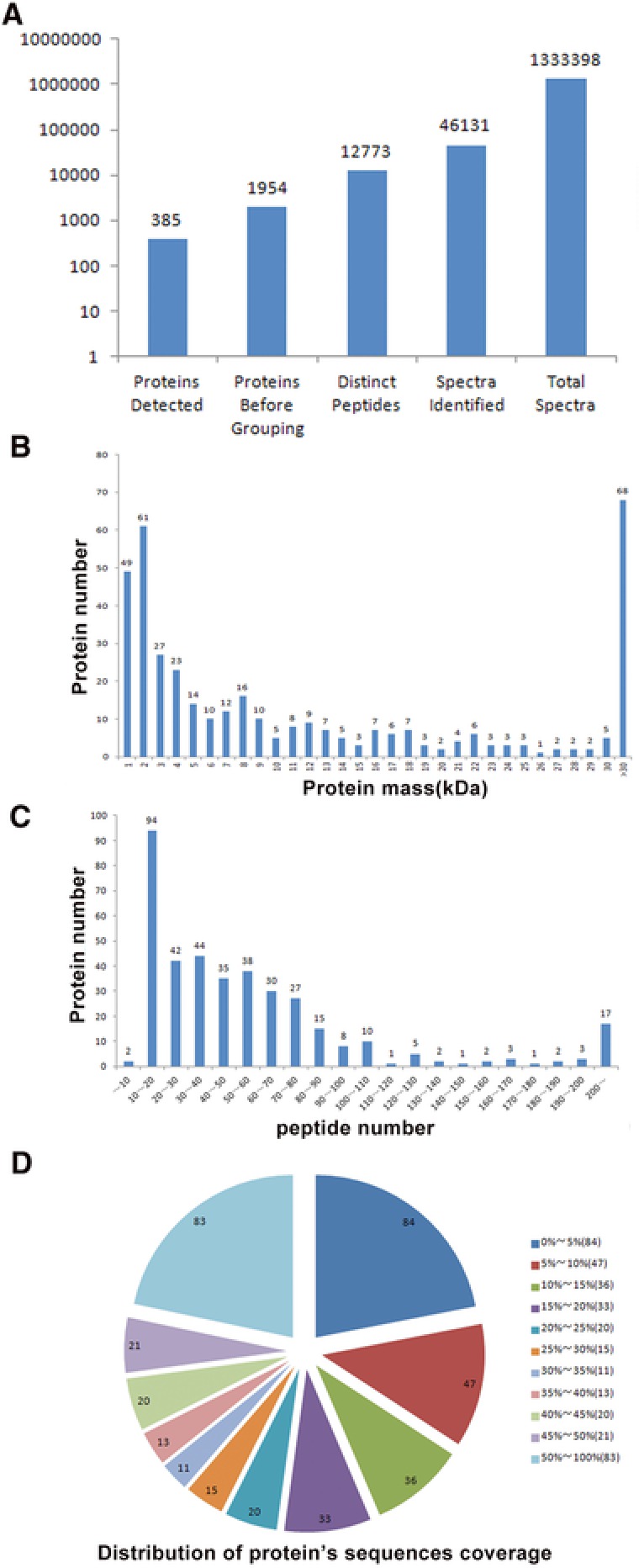
Identification and analysis of the autoimmune cirrhosis proteome. A) Total spectra, spectra identified, distinct peptides, proteins before grouping, and proteins detected from iTRAQ proteomic analysis. B) Protein numbers were grouped based on protein mass. C) Protein numbers were distinguished on the basis of peptide numbers. D) The identified proteins were classified into pie charts according to the protein's sequence coverage.

The goal of this study was to look for potential biomarkers for diagnosis of AIC using iTRAQ. A total of 133 398 spectra, 46 131 spectra identified, 12 773 distinct peptides, 1954 proteins before grouping, and 385 proteins were acquired through the analysis with ProteinPilot Software 5.0.1 search engine (Figure 2A).There were 217, 118, 19, and 28 proteins with a mass of 10–50 kDa, 50–100 kDa, 100–150 kDa, and more than150 kDa, respectively (Figure 2B). The proteins with 1–10 peptides, 11–20 peptides, 21–30 peptides, and above 30 peptides consisted of 227, 57, 31, and 68 proteins, respectively (Figure 2C). Protein sequence coverage with 50–100%, 30–50%, 10–30%, and under 10% variation accounted for 83, 65, 104, and 131, respectively (Figure 2D).

### 3.2 108 Proteins Were Altered between Autoimmune Cirrhosis and Control

Compared to control, a total of 108 differentially expressed proteins were identified in the AIC, these proteins met two conditions, including *p* values ≤ 0.05 and an FDR of less than 1%. Thirty‐two proteins increased by more than1.5‐fold and 76 proteins decreased to less than 0.66‐fold. Detailed information about differential expression proteins is listed in Table 1.

**Table 1:** Expressions of proteins between autoimmune cirrhosis and its normal control

**N**	**Accession no**.	**Protein name**	**protein coverage [%]**	**Peptides confidence (95%)**	**Coefficient of variation**	**Ratio**	***p*‐value**
**Immunoglobulin‐related proteins**							
1	sp|P01857|IGHG1	Ig gamma‐1 chain C region	96.67	243	0.24	4.17	0.011649
2	sp|P01871|IGHM	Ig mu chain C region	74.12	67	0.11	8.07	0.000889
3	sp|B9A064|IGLL5	Immunoglobulin lambda‐like polypeptide 5	55.61	55	0.07	1.61	0.002092
4	sp|P01833|PIGR	Polymeric immunoglobulin receptor	22.12	9	0.10	8.81	0.000581
5	sp|P01591|IGJ	Immunoglobulin J chain	66.04	16	0.12	2.38	0.003724
6	sp|P01860|IGHG3	Ig gamma‐3 chain C region	96.02	100	0.17	4.11	0.004568
7	sp|A0A0B4J1V0|HV315	Immunoglobulin heavy variable 3–15	75.63	12	0.09	1.80	0.003283
8	sp|A0A0B4J1Y8|LV949	Immunoglobulin lambda variable 9–49	39.84	3	0.24	1.94	0.040546
9	sp|P0CG05|LAC2	Ig lambda‐2 chain C regions	96.23	55	0.11	2.13	0.003149
10	sp|P01825|HV459	Immunoglobulin heavy variable 4–59	46.55	8	0.07	1.83	0.001417
11	sp|A0A0B4J1U7|HV601	Immunoglobulin heavy variable 6–1	36.36	4	0.06	1.73	0.001060
12	sp|P01714|LV319	Immunoglobulin lambda variable 3–19	83.04	6	0.15	1.63	0.021422
13	sp|P01701|LV151	Immunoglobulin lambda variable 1–51	66.67	12	0.18	2.12	0.014961
**Complement**							
14	sp|P08603|CFAH	Complement factor H	49.31	52	0.03	−2.85	0.000002
15	sp|P00751|CFAB	Complement factor B	63.61	66	0.17	−2.46	0.000636
16	sp|P01031|CO5	Complement C5	31.92	31	0.01	−2.01	0.000001
17	sp|P10643|CO7	Complement component C7	49.23	33	0.05	2.38	0.000211
18	sp|P13671|CO6	Complement component C6	27.52	23	0.03	−2.29	0.000008
19	sp|P05156|CFAI	Complement factor I	42.88	18	0.02	−1.89	0.000003
20	sp|P06681|CO2	Complement C2	34.84	20	0.12	−1.76	0.001603
21	sp|P04003|C4BPA	C4b−binding protein alpha chain	41.04	22	0.09	−2.02	0.000322
22	sp|P07358|CO8B	Complement component C8 beta chain	25.89	10	0.30	−2.58	0.002908
23	sp|Q03591|FHR1	Complement factor H‐related protein 1	25.45	8	0.11	−1.53	0.003152
24	sp|O43866|CD5L	CD5 antigen‐like	50.14	13	0.07	3.08	0.000539
25	sp|O75636|FCN3	Ficolin‐3	22.74	3	0.08	−1.69	0.000703
26	sp|P02741|CRP	C‐reactive protein	19.64	4	0.10	3.58	0.001164
27	sp|P07333|CSF1R	Macrophage colony‐stimulating factor 1 receptor	7.92	1	0.12	1.65	0.010632
28	sp|P07225|PROS	Vitamin K‐dependent protein S	34.02	19	0.21	−1.87	0.005370
29	sp|P04004|VTNC	Vitronectin	44.56	22	0.10	−2.62	0.000103
**Apolipoprotein**							
30	sp|P04114|APOB	Apolipoprotein B‐100	55.53	231	0.01	−2.58	0.000000
31	sp|P02647|APOA1	Apolipoprotein A1	92.88	124	0.07	−4.29	0.000005
32	sp|P02649|APOE	Apolipoprotein E	76.34	27	0.01	−3.45	0.000005
33	sp|O14791|APOL1.	Apolipoprotein L1	55.28	14	0.14	−1.99	0.001081
34	sp|O95445|APOM	Apolipoprotein M	55.32	6	0.14	−3.41	0.000087
35	sp|P02654|APOC1	Apolipoprotein C‐I	53.01	5	0.08	−2.30	0.000116
36	sp|Q13790|APOF	Apolipoprotein F	16.56	2	0.03	−1.57	0.000064
37	sp|P55056|APOC4	Apolipoprotein C‐IV	8.66	1	0.06	−1.56	0.000404
38	sp|P08519|APOA	Apolipoprotein(a)	18.87	4	0.05	−2.89	0.000006
39	sp|P04114|APOB	Apolipoprotein B‐100	55.53	231	0.01	−2.58	0.000000
40	sp|P10909|CLUS	Clusterin	54.79	35	0.22	−3.44	0.000320
**Haptoglobin**							
41	sp|P00738|HPT	Haptoglobin	90.64	136	0.16	−1.71	0.004644
41	sp|P00739|HPTR	Haptoglobin‐related protein	84.77	56	0.15	−2.29	0.000658
**Serum amyloid**							
43	sp|P02743|SAMP	Serum amyloid P‐component	44.84	12	0.26	−2.95	0.000938
44	sp|P35542|SAA4	Serum amyloid A‐4 protein	55.38	5	0.07	−4.85	0.000003
**Enzymes**							
45	sp|Q14624|ITIH4	Inter‐alpha‐trypsin inhibitor heavy chain H4	76.45	107	0.21	−2.22	0.002157
46	sp|P19823|ITIH2	Inter‐alpha‐trypsin inhibitor heavy chain H2	55.39	82	0.16	−2.36	0.000667
47	sp|P19827|ITIH1	Inter‐alpha‐trypsin inhibitor heavy chain H1	54.34	64	0.26	−3.79	0.000333
48	sp|Q06033|ITIH3	Inter‐alpha‐trypsin inhibitor heavy chain H3	46.97	30	0.15	1.58	0.023043
49	sp|P00747|PLMN	Plasminogen	63.33	61	0.06	−2.40	0.000027
50	sp|P29622|KAIN	Kallistatin	59.72	19	0.25	−2.75	0.001194
51	sp|P27169|PON1	Serum paraoxonase/arylesterase 1	67.32	25	0.17	−2.73	0.000372
52	sp|P36955|PEDF	Pigment epithelium‐derived factor	55.98	21	0.07	−1.77	0.000274
53	sp|P03952|KLKB1	Plasma kallikrein	37.46	14	0.15	−2.56	0.000409
54	sp|Q96PD5|PGRP2	N‐acetylmuramoyl‐L‐alanine amidase	55.21	17	0.32	−2.09	0.009254
55	sp|P05154|IPSP	Plasma serine protease inhibitor	27.34	11	0.35	−4.04	0.000650
56	sp|Q04756|HGFA	Hepatocyte growth factor activator	14.50	7	0.09	−2.22	0.000170
57	sp|P15169|CBPN	Carboxypeptidase N catalytic chain	21.62	6	0.09	−2.13	0.000249
58	sp|P32119|PRDX2	Peroxiredoxin‐2	39.39	8	0.11	1.67	0.008166
59	sp|P04180|LCAT	Phosphatidylcholine‐sterol acyltransferase	23.64	7	0.10	−1.53	0.002907
60	sp|Q96KN2|CNDP1	Beta‐Ala‐His dipeptidase	30.37	9	0.05	−2.00	0.000070
61	sp|P06276|CHLE	Cholinesterase	24.09	7	0.18	−3.86	0.000108
62	sp|Q96IY4|CBPB2	Carboxypeptidase B2	23.17	7	0.14	−1.71	0.003235
63	sp|P00918|CAH2	Carbonic anhydrase 2	32.31	5	0.09	2.14	0.001921
64	sp|Q92820|GGH	Gamma‐glutamyl hydrolase	25.79	3	0.05	−1.89	0.000075
65	sp|P08294|SODE	Extracellular superoxide dismutase [Cu‐Zn]	10.42	2	0.06	−1.54	0.000547
66	sp|P80108|PHLD	Phosphatidylinositol‐glycan‐specific phospholipase D	33.69	22	0.20	−1.96	0.003617
**Glycoprotein**							
67	sp|P08697|A2AP	Alpha‐2‐antiplasmin	65.78	41	0.09	−1.64	0.000003
68	sp|P25311|ZA2G	Zinc‐alpha‐2‐glycoprotein	78.19	28	0.04	−1.59	0.001300
69	sp|P02763|A1AG1	Alpha‐1‐acid glycoprotein 1	68.66	62	0.07	1.98	0.000216
70	sp|P02750|A2GL	Leucine‐rich alpha‐2‐glycoprotein	54.76	19	0.11	1.69	0.002183
71	sp|P19652|A1AG2	Alpha‐1‐acid glycoprotein 2	64.68	29	0.17	−1.83	0.001083
72	sp|P12814|ACTN1	Alpha‐actinin‐1	8.97	2	0.16	2.80	0.007323
73	sp|P02671|FIBA	Fibrinogen alpha chain	67.21	209	0.09	−1.64	0.005676
74	sp|P02765|FETUA	Alpha‐2‐HS‐glycoprotein	75.48	77	0.03	−3.54	0.000020
75	sp|P02751|FINC	Fibronectin	41.70	80	0.12	−1.53	0.000105
76	sp|P02790|HEMO	Hemopexin	84.42	90	0.07	−5.33	0.000009
77	sp|P05546|HEP2	Heparin cofactor 2	64.13	44	0.12	−3.48	0.000009
78	sp|P02749|APOH.	Beta‐2‐glycoprotein 1	53.91	16		−3.23	0.000070
**Hemoglobin**							
79	sp|P69891|HBG1	Hemoglobin subunit gamma‐1	74.15	16	0.10	2.02	0.002997
80	sp|P68871|HBB	Hemoglobin subunit beta	99.32	56	0.11	2.09	0.003617
81	sp|P02042|HBD	Hemoglobin subunit delta	99.32	42	0.02	1.51	0.000081
**Insulin‐like growth factor**							
82	sp|P35858|ALS	Insulin‐like growth factor‐binding protein complex acid labile subunit	43.80	17	0.41	−3.32	0.000016
83	sp|P17936|IBP3	Insulin‐like growth factor‐binding protein 3	28.52	4	0.08	−1.51	0.001804
**Coagulation factor**							
84	sp|P01042|KNG1	Kininogen‐1	63.04	73	0.09	−1.49	0.002136
85	sp|P00734|THRB	Prothrombin	65.11	46	0.07	−2.28	0.000063
86	sp|P04196|HRG	Histidine‐rich glycoprotein	58.86	40	0.18	−3.35	0.000179
87	sp|P00748|FA12	Coagulation factor XII	38.21	18	0.28	−1.69	0.024572
88	sp|P04275|VWF	von Willebrand factor	39.74	12	0.14	3.02	0.003669
89	sp|P05160|F13B	Coagulation factor XIII B chain	30.26	12	0.10	−1.58	0.002278
90	sp|P12259|FA5	Coagulation factor V	11.33	9	0.30	−1.85	0.016346
91	sp|P00740|FA9	Coagulation factor IX	26.03	8	0.05	−1.61	0.000171
92	sp|P04070|PROC	Vitamin K‐dependent protein C	18.87	8	0.06	−1.61	0.000431
**Unclassified**							
93	sp|P02774|VTDB	Vitamin D‐binding protein	76.58	65	0.11	−3.74	0.000030
94	sp|P02766|TTHY	Transthyretin	86.39	72	0.30	−3.94	0.000455
95	sp|P18206|VINC	Vinculin	15.26	9	0.06	1.62	0.001495
96	sp|P05452|TETN	Tetranectin	57.92	18	0.08	−1.82	0.000391
97	sp|P19320|VCAM1	Vascular cell adhesion protein 1	24.36	9	0.07	1.99	0.001162
98	sp|P28676|GRAN	Grancalcin	12.44	3	0.03	1.85	0.000095
99	sp|P49908|SEPP1	Selenoprotein P	19.69	5	0.05	−2.55	0.000014
100	sp|P02760|AMBP	Protein AMBP	49.43	24	0.10	−1.98	0.000495
101	sp|Q92954|PRG	Proteoglycan 4	8.55	8	0.07	−1.65	0.000616
102	sp|P06396|GELS	Gelsolin	65.60	56	0.19	−7.28	0.000011
103	sp|P01019|ANGT	Angiotensinogen	55.26	47	0.09	−2.62	0.000084
104	sp|P05543|THBG	Thyroxine‐binding globulin	57.83	32	0.27	−4.11	0.000262
105	sp|P22792|CPN2	Carboxypeptidase N subunit 2	34.50	13	0.07	−1.52	0.000955
106	sp|Q16610|ECM1	Extracellular matrix protein 1	26.11	7	0.03	−1.78	0.000031
107	sp|Q15848|ADIPO	Adiponectin	28.69	8	0.03	2.79	0.000063
108	sp|P60709|ACTB	Actin, cytoplasmic 1	57.07	21	0.06	1.69	0.001240

### 3.3 Classification of Identified Proteins

**Figure 3:**
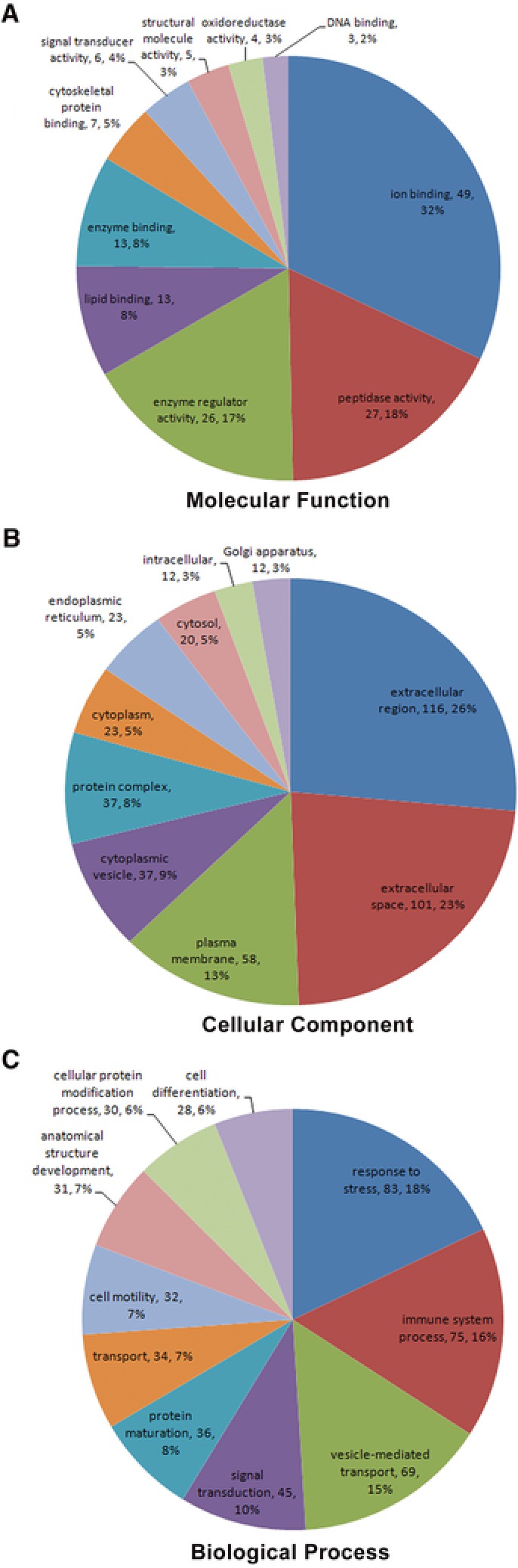
GO assignment of differential expression of proteins in autoimmune cirrhos**is**. A) Molecular function. B) Cellular component. C) Biological processes.

According to GO classification system, differentially expressed proteins were distributed into categories based on molecular function, cellular components, and biological processes. For molecular functions, 32% proteins were related to response to ion binding, followed by peptidase activity (18%), enzyme regulator activity (17%), and lipid binding (8%; Figure 3A). For cellular components, 26% were related to extracellular region and 23% to extracellular space, followed by plasma membrane (13%) and cytoplasmic vesicle (9%; Figure 3B).Stress represented 18% of the biological processes, followed by immune system process (16%), vesicle‐mediated transport (15%), and signal transduction (10%; Figure 3C).

### 3.4 KEGG Pathway Analysis of Identified Proteins

As shown in Table 2, the 108 differential expression proteins were further investigated using the KEGG database, and they were found to be enriched in complement and coagulation cascades (14.8%), *Staphylococcus aureus* infection (4.4%), systemic lupus erythematosus (3.8%), regulation of actin cytoskeleton (3.3%), focal adhesion (3.3%), and African trypanosomiasis (2.7%).

**Table 2:** KEGG enrichment analysis of differential expression of proteins

**KEGG pathway**	**Number of proteins**	**Pathway ID**
Complement and coagulation cascades	27	hsa04610
Staphylococcus aureus infection	8	hsa05150
Systemic lupus erythematosus	7	hsa05322
Regulation of actin cytoskeleton	6	hsa04810
Focal adhesion	6	hsa04510
African trypanosomiasis	5	hsa05143
Amoebiasis	4	hsa05146
Prion diseases	4	hsa05020
Leukocyte transendothelial migration	4	hsa04670
PI3K‐Akt signaling pathway	4	hsa04151

### 3.5 Protein–Protein Interaction Network

**Figure 4:**
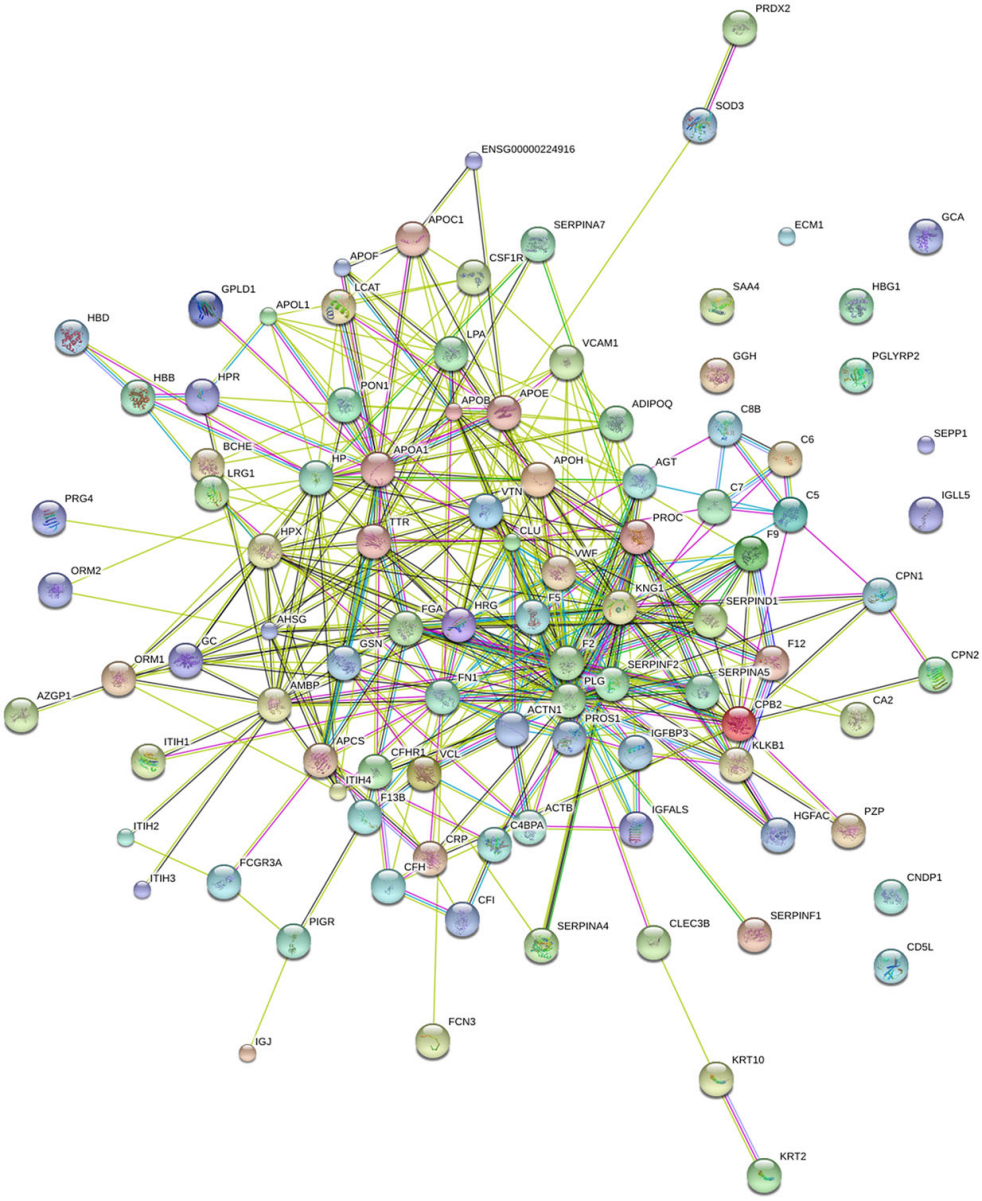
Interaction network analysis of differential expression of proteins. In this network, nodes are proteins, lines represent functional associations between proteins, and different line colors represent the types of evidence for the predicted functional association. A red line indicates the presence of fusion evidence; a green line indicates neighborhood evidence; a blue line indicates co‐ocurrence evidence; a purple line indicates experimental evidence; a yellow line indicates text mining evidence; a light blue line indicates database evidence; a black line indicates coexpression evidence.

Biological systems can be regulated as complex network systems with many interactions among the components in different pathways. In order to better understand the pathogenic mechanisms in AIC, the protein interaction network for the identified variable proteins was constructed by STRING. From the network diagram (Figure 4), many proteins were at the core of the “traffic link,” such as vWF, IFGALS, apoA1, apoE, and so on, which suggest that they may play an important role in the development of AIC.

### 3.6 Validation of Candidate Biomarkers Using Western Blot

**Figure 5:**
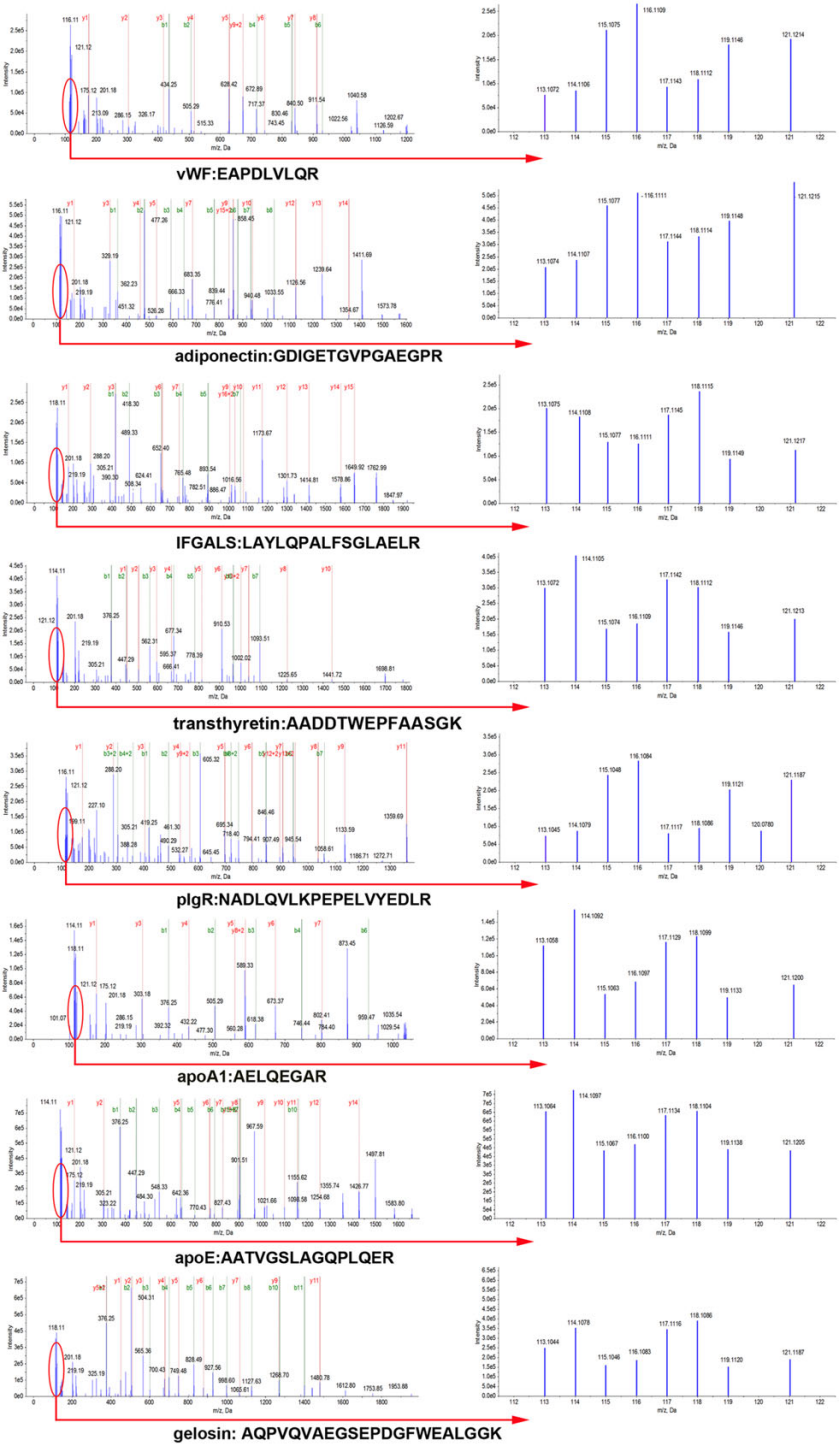
MS/MS spectrum to show the iTRAQ quantification. A representative MS/MS spectrum showed peptide signatures for vWF, adiponectin, IFGALS, transthyretin, pIgR, apoA1, apoE, and gelsolin. Ratios of iTRAQ tags indicate the relative abundance of the eight proteins individually in autoimmune cirrhosis serum compared to corresponding control samples.

**Figure 6:**
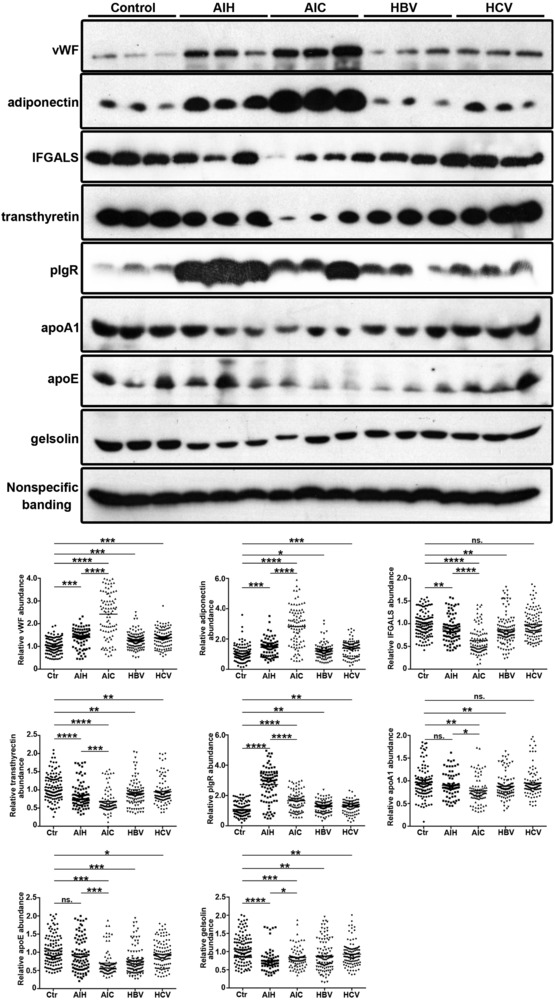
Western blot validation of selected proteins in the iTRAQ data set. Representative western blots for three proteins validated in serum with vWF, adiponectin, IFGALS, pIgR, transthyretin, apoA, apoE, and gelsolin. Data are expressed as the mean. Control (*n* = 120), AIH (*n* = 90), AIC (*n* = 90), HBV (*n* = 120), HCV (*n* = 120). *p < 0.05, **p < 0.01, ***p < 0.001, ****p < 0.0001.

The 108 proteins which were identified using iTRAQ were classified into ten categories, based on protein function and eight significant proteins (including apoA1, apoE, vWF, IFGALS, gelsolin, adiponectin, TTR, pIgR) were verified further using western blot (Table 3), their MS/MS spectrogram were added to show the iTRAQ quantification (Figure 5). Western blot results showed that compared with the normal controls, the ratio of vWF, adiponectin, pIgR was, respectively, increased 2.43 (*p* < 0.0001), 2.82 (*p* < 0.0001), 1.66 (*p* < 0.0001) in AIC, 1.39 (*p* < 0.001), 1.46 (*p* < 0.001), 2.72 (*p* < 0.0001) in AIH, 1.27 (*p* < 0.001), 1.21 (*p* < 0.05), 1.28 (*p* < 0.01) in HBV and 1.38 (*p* < 0.001), 1.40 (*p* < 0.001), 1.30 (*p* < 0.01) in HCV. However, the ratio of IFGALS, TTR, apoA1, apoE, gelsolin was, respectively, decreased to 0.63 (*p* < 0.0001), 0.68 (*p* < 0.0001), 0.78 (*p* < 0.01), 0.70 (*p* < 0.001), 0.83 (*p* < 0.001) in AIC, 0.89 (*p* < 0.01), 0.85 (*p* < 0.0001), 0.90 (*p* > 0.05), 0.95 (*p* > 0.05), 0.75 (*p* < 0.0001) in AIH, 0.86 (*p* < 0.001), 0.90 (*p* < 0.01), 0.88 (*p* < 0.01), 0.75 (*p* < 0.001), 0.88 (*p* < 0.01) in HBV and 0.98 (*p* > 0.05), 0.95 (*p* < 0.01), 0.95 (*p* > 0.05), 0.94 (*p* < 0.05), 0.93 (*p* < 0.01) in HCV (Figure 6).Western blot results showed the same trend of change with iTRAQ methods in AIC, which confirmed the reliability of the iTRAQ results.

**Table 3:** Categories of significant differential expression of proteins in autoimmune cirrhosis

N	Accession no.	Protein name	Ratio	*p*‐value	Function
1	sp|P04275|VWF	von Willebrand factor	3.02	0.003669	Hypercoagulability and thrombosis
2	sp|Q15848|ADIPO	Adiponectin	2.79	0.000063	Insulin‐sensitizing adipokine
3	sp|P35858|ALS	Insulin‐like growth factor‐binding protein complex acid labile subunit	−3.32	0.000016	Increasing IGF half‐life and vascular localization
4	sp|P02766|TTHY	Transthyretin	−3.94	0.000455	Transport of thyroxine and retinol
5	sp|P01833|PIGR	Polymeric immunoglobulin receptor	8.81	0.000581	Transport of pIg (particular dimeric IgA)
6	sp|P02647|APOA1	Apolipoprotein AI	−4.29	0.000005	Lipoprotein metabolism
7	sp|P02649|APOE	Apolipoprotein E	−3.45	0.000005	Lipoprotein metabolism
8	sp|P06396|GELS	Gelsolin	−7.28	0.000011	Actin regulator

### 3.7 ROC Curve Analysis

**Figure 7:**
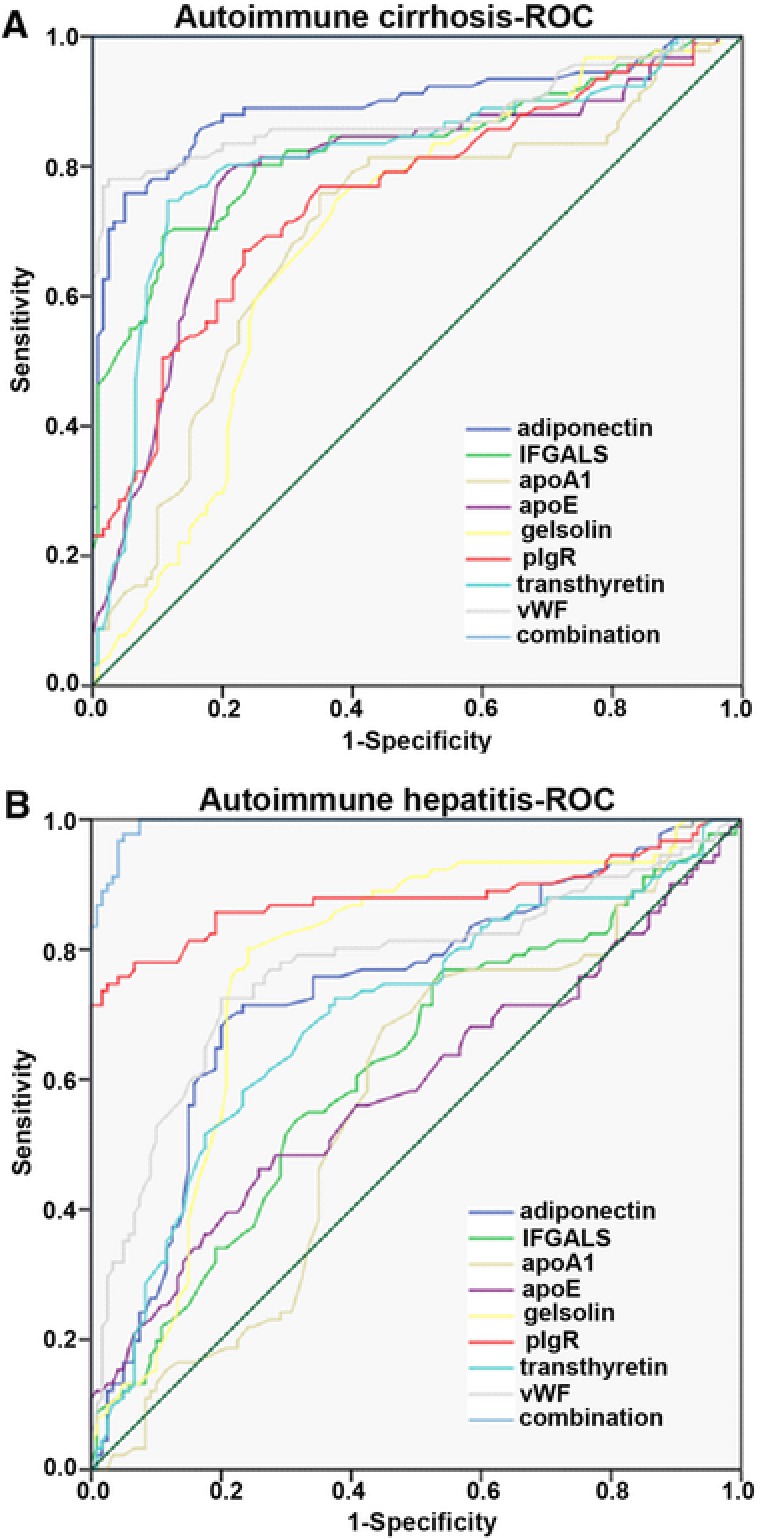
Receiver operating characteristic (ROC) curves for selected proteins in autoimmune cirrhosis and autoimmune hepatitis. ROC analysis for vWF alone, adiponectin alone, IFGALS alone, pIgR alone, transthyretin alone, apoA alone, apoE alone, gelsolin alone, and their combination in the diagnosis of AIC and AIH.

To evaluate the specificity and sensitivity of each marker and their combination in the diagnosis of AIC and AIH, we conducted ROC analysis. The accuracy of the area under the ROC curve was assessed as follows: 0.9–1 = excellent, 0.8–0.9 = good, 0.7–0.8 = fair, 0.6–0.7 = poor, and <0.6 = not useful. In AIC, ROC analysis results are as follows: vWF (AUC: 0.880; 95% CI 0.825–0.934), adiponectin (AUC: 0.895; 95% CI 0.845–0.944), pIgR (AUC: 0.757; 95% CI 0.690–0.824), IFGALS (AUC: 0.829; 95% CI 0.769–0.888), TTR (AUC: 0.814; 95% CI 0.750–0.877), apoA1 (AUC: 0.700; 95% CI 0.627–0.773), apoE (AUC: 0.788; 95% CI 0.722–0.854), gelsolin (AUC: 0.697; 95% CI 0.626–0.768). The combination of these eight proteins displays a significant predictive value for AIC (AUC: 1.000; 95% CI 1.000–1.000) (Figure 7A). In AIH, ROC analysis results are as follows: vWF (AUC: 0.770; 95% CI 0.702–0.839), adiponectin (AUC: 0.737; 95% CI 0.668–0.807), pIgR (AUC: 0.880; 95% CI 0.824–0.936), IFGALS (AUC: 0.617; 95% CI 0.540–0.694), TTR (AUC: 0.697; 95% CI 0.624–0.771), apoA1 (AUC: 0.567; 95% CI 0.488–0.645), apoE (AUC: 0.587; 95% CI 0.506–0.667), gelsolin (AUC: 0.770; 95% CI 0.704–0.837). The combination of these eight proteins also displays a significant predictive value for AIH (AUC: 0.994; 95% CI 0.989–1.000; Figure 7B).

## 4 Discussion

AIC seriously impairs human's life and health. Once the clinical symptoms appear, it has changed to late stage with many complications which seriously endanger the lives of patients, resulting in missing the best time for treatment. Therefore, early diagnosis is necessary for assessing the risk of progression and planning the appropriate treatment. Unfortunately, we lack the means for early diagnosis at the present stage.

In the present study, we took advantage of iTRAQ‐based proteomics which has the advantages of adequate sensitivity, high reproducibility, and wide linear dynamic range over traditional proteomics,^14^ to identify diagnostic markers of AIC. The majority of the differentially expressed proteins are involved in the immunoglobulin system, complement activation system, lipid metabolism system, and coagulation system.

vWF's primary function is binding to factor VIII, which is important in platelet adhesion to wound sites, so vWF plays a major role in blood coagulation.^15^ Recent research suggests that vWF is also involved in the formation of blood vessels themselves, which would explain why some people with vWF disease develop vascular malformations that can bleed excessively.^16^ The biological breakdown of vWF is largely mediated by the enzyme ADAMTS13 which is synthesized mainly in liver cells,^17^ so the elevation of vWF may be involved in vascular remodeling leading to gastrointestinal bleeding tendency (one of the most serious complications) in AIC. Compared with the control group, vWF increased to 1.39 times in AIH and continued to increase to 2.43 times in AIC. However, there was relatively low expression in HBV and HCV. Meanwhile, ROC analysis (AUC: 0.880; 95% CI 0.825–0.934) showed that vWF could be a better indicator of AIC, and was also a better dynamic observation index in different stages of autoimmune liver disease.

Adiponectin is the major insulin‐sensitizing adipokine, mainly secreted by hepatocytes and adipocytes. Adiponectin exhibits anti‐inflammatory, anti‐hyperglycemic, and anti‐atherogenic properties, which could be beneficial in the prevention and treatment of disorders associated with overweight.^18^ Adiponectin also plays a pivotal role in metabolic liver disease, it exerts direct effects on hepatocytes,^19^ regulates two metabolic pathways, anti‐inflammatory peroxisome proliferator‐activated receptor α, and fatty acid oxidation, suggesting that adiponectin may potentially play hepatoprotective roles against liver fibrosis and cirrhosis.^20^ Compared with the control group, adiponectin increased to 1.46 times in AIH and continued to rise to 2.82 times in AIC. Meanwhile, ROC analysis (AUC: 0.895; 95% CI 0.845–0.944) showed that adiponectin could be a better indicator of AIC, and was also a better dynamic observation target in different stages of autoimmune liver disease.

IGFALS is synthesized mostly by the plasma cells of the liver. IGFALS is able to combine with insulin‐like growth factors (IGFs) to form a heterologous protein complex, which can prolong the half‐life of IGFs in the blood.^21^ AIC resulted in a significant decrease in the synthesis of IGFALS, which may be due to downregulation expression of the growth hormone receptor or due to an increase of growth hormone rejection. Comparing with the control group, it is shown that IGFALS dropped to 0.89 in AIH, and later went on to decrease to 0.63 in AIC. Besides, ROC analysis (AUC: 0.829; 95% CI 0.769–0.888) reflected that IGFALS could be a better indicator for AIC.

Autoimmune hepatitis (AIH) is one of the autoimmune diseases and is characterized by increases in the levels of γ‐globulins and immunoglobulin G in the blood.^22^ The experimental results showed different subtypes of immunoglobulin and immunoglobulin receptor increased in different degrees in the serum. Polymeric immunoglobulin receptor (pIgR) is a transmembrane glycoprotein, selectively expressed by mucosal and glandular epithelial cells. pIgR mediates transport of pIg (in particular dimeric IgA) into external secretions, which provide the first line of adaptive immune defense against ingested, inhaled, and sexually transmitted pathogens.^23^ The key regulator of pIgR expression is cytokines, such as INF‐γ, TNF, and IL‐1.^24^ In comparison with the control group, pIgR increased to 2.7 times in AIH and lowered to 1.66 times in AIC. ROC analysis (AUC: 0.880; 95% CI 0.824–0.936) showed that pIgR could be a better indicator of AIH as well as a better dynamic observation index in different courses of the autoimmune liver disease.

TTR, known as prealbumin, has a major role in the transport of thyroxine and retinol as a transporter.^25^ Serum TTR might be a sensitive indicator in assessing liver dysfunction in acute liver diseases.^26^ Transferrin was also reduced in chronic hepatitis C and hepatitis B.^27^ Compared to the control group, TTR dropped to 0.85 in AIH, and later continued to decrease to 0.68 in AIC. In the meanwhile, ROC analysis (AUC: 0.814; 95% CI 0.750–0.877) obviously indicated that TTR could be a good indicator of AIC.

Apolipoprotein is mostly synthesized in the liver,^28^ which plays an important role in lipoprotein metabolism, including five subtypes apoA, apoB, apoC, apoD, apoE. apoE transports lipoproteins, fat‐soluble vitamins, and cholesterol into the lymph system and then into the blood. apoE was initially recognized for its importance in lipoprotein metabolism and cardiovascular disease. More recently, it has been studied for its role in several biological processes, including Alzheimer's disease (AD),^29^ immunoregulation, and cognition.^30^ In the field of immune regulation, a growing number of studies point to apoE's interaction with many immunological processes, including suppressing T cell proliferation, macrophage functioning regulation, lipid antigen presentation facilitation (by CD1) to natural killer T cell as well as modulation of inflammation and oxidation.^31^ apoA1 is the major protein component of HDL particles, apoA1 (the ratio apoB‐100/apoA1) is often used as a biomarker for prediction of cardiovascular diseases.^32^ The extent of liver damage is directly proportional to the low level of apoE and apoA1. So serum apolipoprotein can be used as an important indicator to assess the severity of cirrhosis. However, ROC analysis apoA1 (AUC: 0.700; 95% CI 0.627–0.773), apoE (AUC: 0.788; 95% CI 0.722–0.854) in AIC, apoA1 (AUC: 0.567; 95% CI 0.488–0.645), apoE (AUC: 0.587; 95% CI 0.506–0.667) in AIH showed apoA1 and apoE were not ideal diagnostic indicators.

Gelsolin, a Ca^2+^‐regulated actin filament severing and capping protein, is a highly conserved, polyfunctional regulator of cell structure and metabolism.^33^ Previous research demonstrated that gelsolin was prevalently expressed in a variety of cells and was decreased in various liver diseases,.^34^ However, ROC analysis (AUC: 0.697; 95% CI 0.626–0.768 in AIC and AUC: 0.770; 95% CI 0.704–0.837 in AIH) showed gelsolin was also not an ideal diagnostic indicator.

In the study, we first implemented iTRAQ technique to identify vWF, adiponectin, IFGALS, and TTR as candidate biomarkers in AIC, which will provide a useful basis for further analysis of the pathogenic mechanism.

## Conflict of Interest

The authors declare no conflict of interest.
